# Omadacycline successfully treated severe *Legionella* pneumonia after moxifloxacin treatment failure: Case series

**DOI:** 10.3389/fphar.2025.1559857

**Published:** 2025-03-10

**Authors:** Biyun Li, Laiyu Liu, Fei Li, Chunmei He, Yuan Chen, Xiaodong Tian, Zhenyu Liu, Weizhen Zhang

**Affiliations:** ^1^ Department of Respiratory and Critical Care Medicine, Nanfang Hospital, Southern Medical University, Guangzhou, China; ^2^ Department of Pharmacy, The Second People’s Hospital of Futian District, Shenzhen, China; ^3^ Department of Pharmacy, Shenzhen Nanshan People’s Hospital, Shenzhen, China

**Keywords:** omadacycline, *Legionella pneumophila*, susceptibility, moxifloxacin, case series

## Abstract

*Legionella* is a significant pathogen responsible for community-acquired pneumonia and, less commonly, for hospital-acquired pneumonia. *Legionella pneumophila*, the most prevalent species within the *Legionella* genus, accounts for 80%–90% of human infections, and often leads to severe pneumonia complicated by multi-organ dysfunction. Omadacycline, a novel tetracycline, has demonstrated *in vitro* activity against atypical pathogens, including *L. pneumophila*; however evidence regarding its application in severe *Legionella* pneumonia remains limited. In this paper, we report 3 cases of successful treatment of severe *Legionella* pneumonia with omadacycline in patients who initially did not respond to empirical treatment with moxifloxacin, aiming to provide clinical experience and guidance for the use of omadacycline.

## 1 Introduction

*Legionella* disease is a systemic disease caused by *Legionella* infection, primarily characterized by pneumonia and often accompanied by high fever, mental disorders, myalgia and diarrhea, which can rapidly progress to severe pneumonia, frequently leading to multiple organ damage (Iliadi et al, 2022). *Legionella* is the most prevalent pathogen associated with atypical pneumonia in hospitalized patients, accounting for 2%–9% of cases (Cunha et al., 2016). Up to 44% of hospitalized patients with this infection may require admission to the intensive care unit (ICU), ranking it second only to *Streptococcus* pneumoniae and Enterobacteriaceae, with a mortality rate ranging from 10% to 15% (Dooling et al., 2015; Brown, 2004; Qu et al., 2022). Omadacycline, a novel aminomethylcycline broad-spectrum antibiotic, has been minimally reported as a second-line treatment for severe *Legionella pneumophila* pneumonia (Markham and Keam, 2018; Wang et al., 2024). This paper reports three patients with severe *Legionella* pneumonia who were successfully treated with omadacycline after initially failing to respond to empirical treatment with moxifloxacin.

## 2 Case presentation

### 2.1 Case 1

**FIGURE 1:**
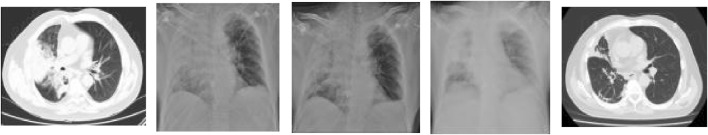
Images data acquired overtime showing changes associated with therapeutic interventions. **(A)**: on arrival, chest CT showed infectious lesions in the right lung; **(B–D)**: before the treatment, bilateral lung lesions progressed on chest X-ray; **(E)**: after treatment, significant improvement was revealed, chest CT scans at discharge.

A 58-year-old male was admitted to the hospital on 27 August 2024, due to a cough and sputum production persisting for 1 week, along with chest pain for 5 days. The patient initially developed symptoms in the community, characterized by coughing up yellow phlegm, occasionally mixed with blood, and experiencing chest pain during violent coughing episodes. He also reported fatigue and shortness of breath, which became more pronounced after activity. Despite receiving symptomatic treatment at a local hospital (unknown drugs used), his condition did not improve significantly, prompting his transfer to our center for further treatment. Upon admission, his pulse oxygen saturation was recorded at 90%–92%. Laboratory tests revealed elevated C-reactive protein (CRP 192.65 mg/L; normal range 0.0–6.0 mg/L), procalcitonin (PCT 0.588 ng/mL; normal range 0.00–0.05 ng/mL), creatinine (CREA 132 μmol/L; normal range 41–81 μmol/L), and alanine aminotransferase (ALT 77U/L; normal range 7–40U/L). A natural sputum smear showed a white blood cell count of less than 25 per low-power field and fewer than 10 epithelial cells per low-power field, with a few Gram-positive cocci and Gram-negative bacilli identified. Chest computed tomography (CT) imaging showed infectious lesions in the right lung (Figure 1A). The patient was initially diagnosed with severe pneumonia and was empirically treated with meropenem, administered as a single intravenous infusion of 0.5 g every 8 h. On August 29, the patient continued to experience cough and produced a large volume of yellow sputum, with inflammatory markers showing further elevation: white Blood Cell (WBC) count of 17.6*10^9^/L (normal range 3.50–9.50*10^9^/L), CRP at 223.89 mg/mL, and PCT at 0.581 ng/mL. The imaging indicated that the infectious lesions were more advanced (Figure 1B). Given the patient’s critical condition, characterized by severe pneumonia complicated by renal insufficiency, Teicoplanin was added to his treatment regimen (first dose: 0.4 g intravenously every 12h, followed by: 0.4 g/d). On September 1, the patient developed a fever (maximum temperature (Tmax) of 38.4°C), with no significant improvement in inflammatory markers, and atypical pathogens were not excluded. Moxifloxacin (single intravenous infusion of 0.4 g/d) was subsequently administered, and the dosage of meropenem was adjusted to 1.0 g every 8 h. On September 4th, analysis of a bronchoalveolar lavage fluid (BALF) sample using targeted next-generation sequencing (tNGS) detected *L. pneumophila* (sequence 48 reads). Given the patient’s clinical symptoms and imaging characteristics (Figure 1C), a diagnosis of *Legionella* infection was established, leading to the discontinuation of teicoplanin. On September 8, the patient continued to experience shortness of breath with exertion, inflammatory indicators increased, and chest X-ray revealed progression of bilateral lung lesions (Figure 1D). Considering the potential for moxifloxacin-resistant *Legionella*, the treatment was switched from moxifloxacin to omadacycline (first dose of 300 mg every 12 h administered orally, followed by 300 mg/d). After treatment with omadacycline, the patient’s respiratory symptoms significantly improved. On September 15th, a chest CT re-examination indicated resolution of the bilateral lung lesions (Figure 1E), and the patient was discharged in stable condition.

### 2.2 Case 2

**FIGURE 2:**
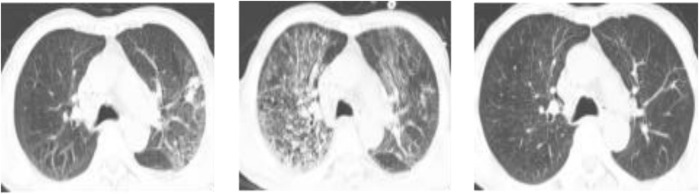
Images of chest CT scans acquired overtime showing changes associated with therapeutic interventions. **(A)**: on arrival, chest CT showed infectious lesions in the left lung; **(B)**: before the treatment, bilateral lung lesions progressed; **(C)**: after treatment, significant improvement was revealed.

A 66-year-old male was diagnosed with ANCA (antineutrophil cytoplasmic antibodies)-associated vasculitis and ANCA-associated vasculitis renal injury for over 3 months, during which he underwent intermittent hemodialysis. He was admitted to the hospital on 24 June 2024, due to intermittent fever with headache lasting more than 3 months and elevated serum creatinine levels for over 1 month. Upon admission, he received symptomatic treatment. On July 4, the patient developed a fever (Tmax 37.8°C) after dialysis, along with intermittent cough and white sputum production. Laboratory results indicated elevated inflammatory markers: WBC at 11.41*10^9^/L, CRP at 53.88 mg/mL, PCT at 0.396 ng/mL, albumin (ALB) at 28.2 g/L (normal range 40.0–55.0 g/L), and CREA at 413 μmol/L. CT imaging showed infectious lesions in the left lung (Figure 2A). The initial anti-infection regimen consisted of moxifloxacin (single intravenous infusion of 0.4 g/d). On July 6, the patient remained febrile (Tmax 38.9°C), and auscultation of both lower lungs revealed scattered moist rales. The tNGS suggested infections with *L. pneumophila* (sequence 306 reads), Pneumocystis yerinii (sequence 89 reads), and cytomegalovirus (sequence 102 reads). Consequently, Trimethoprim/sulfamethoxazole (TMP-SMZ) (0.96 g orally every q8h) was administered. On July 10, the patient continued to experience cough, producing light red sputum, with a decrease in oxygenation index and an increase in inflammation markers: CRP at 68.32 mg/mL, PCT at 1.44 ng/mL, and CREA at 478 μmol/L. Chest CT showed diffuse exudation in both lungs, indicating significant aggravation (Figure 2B); Additionally, the patient exhibited limb twitching and hand shaking, and the adverse reactions caused by moxifloxacin were not ruled out after consultation with the professor of the Department of Pharmacy. Considering the patient’s long-term use of immunosuppressants and inadequate infection control, the anti-infection regimen was adjusted to include omadacycline (initial dose of 300 mg every 12 h administered orally, followed by 300 mg/d), caspofungin (initial dose of 70 mg/d administered intravenously, followed with 50 g/d), and ganciclovir (single intravenous infusion of 0.075 g/d). Following the adjustment of the anti-infection regimen, the patient’s dyspnea improved, and a chest CT re-examination indicated basically resolved (Figure 2C), leading to the patient’s discharge 1 week later.

### 2.3 Case 3

**FIGURE 3:**
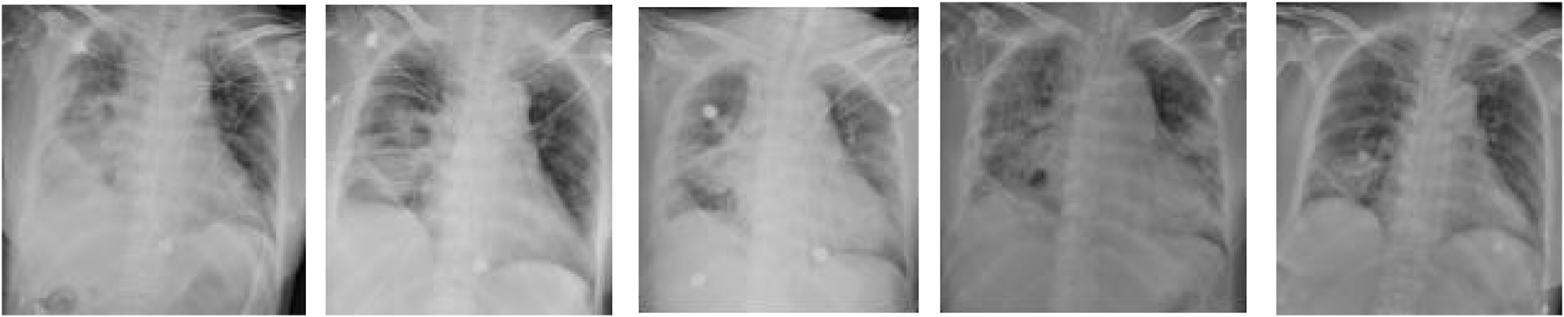
Images of chest x-ray acquired overtime showing changes associated with therapeutic interventions. **(A)**: on arrival, Chest radiograph showed bilateral infectious lung lesions and pleural effusion; **(B–D)**: before the treatment, progressive exudation in both lungs; **(E)**: after treatment, largely resolution was revealed.

A 62-year-old female was diagnosed with ANCA-associated vasculitis and renal injury related to ANCA-associated vasculitis for more than 3 months, during which she underwent intermittent hemodialysis. She was admitted to hospital on 7 October 2022, due to a cough persisting for more than 4 months and confirmed diagnosis of ANCA-related vasculitis. Upon admission, the temperature of this patient was 39.0°C, and coarse respiratory sounds were noted in both lungs, with wet rales heard in the lower right lung. The inflammatory markers were elevated, with a WBC of 6.71*10^9^/L, platelet (PLT) of 9*10^9^/L (normal range 125–350*10^9^/L), CRP at 389.37 mg/L, Total Bilirubin (TBIL) of 29.9 μmol/L (normal range 0.0–21.0 μmol/L), ALB at 23.0 g/L, and CREA of 219 μmol/L, Chest radiography revealed bilateral infectious lung lesions and pleural effusion (Figure 3A). The initial diagnosis was sepsis with lung infection. Given that the patient had a history of recurrent infections over the past year and had been exposed to multiple broad-spectrum antimicrobials, she was empirically treated with meropenem (1.0 g intravenously every 12 h) and teicoplanin (0.4 g intravenously every 48 h). On October 20, the patient’s temperature, inflammatory indicators, and imaging showed improvement (Figure 3B). On October 23, the patient’s temperature remained normal for 3 days, and the treatment regimen was changed to piperacillin/tazobactam (4.5 g intravenously every 8 h) and teicoplanin (0.4 g intravenously every 48 h). However, on November 3, the patient experienced a recurrence of fever (Tmax 38.4°C), and the right lower lung had scattered moist rales. The inflammatory index markers had increased, with a WBC of 4.44*10^9^/L, CRP at 73.92 mg/L, PCT at 4.19 ng/mL, and CREA at 91 μmol/L. Consequently, the anti-infection regimen was switched to meropenem (1.0 g intravenously every 8 h) and caspofungin (initial dose of 70 mg/d intravenously, followed by 50 g/d). On November 4, the patient was still febrile (Tmax 38.7°C) and developed hemoptysis. Chest radiography showed progressive exudation in both lungs (Figure 3C). Blood tNGS indicated the presence of *L. pneumophila* (sequence 32 reads) and human cytomegalovirus (sequence 228 reads). Given the patient’s long-term use of immunosuppressants, alongside the imaging and pulmonary alveolar lavage fluid tNGS results, the anti-infection regimen was adjusted to include meropenem (1.0 g administered intravenously every 8 h), moxifloxacin (0.4 g administered intravenously daily), TMP-SMZ (0.96 g orally every 8 h) and ganciclovir (0.075 g administered intravenously daily). On November 8, the patient developed mental disorders, blood pressure decreased to 87/54 mmHg, and inflammatory markers were elevated: WBC at 10.57*10^9^/L, CRP at 173.51 mg/L, PCT at 0.198 ng/mL, and CREA at 136 μmol/L. Chest X-ray showed rapidly progressing exudative lesions in both lungs (Figure 3D). Septic shock was suspected, and the patient was transferred to the ICU. Alveolar lavage fluid tNGS results indicated *L. pneumophila* (sequence 3,831 reads) and CMV (sequence 4,952 reads). The treatment regimen was further adjusted to include meropenem (1.0 g administered intravenously every 8 h), omadacycline (first dose of orally 300 mg every 12h, followed by 300 mg/d), caspofungin (first dose of 70 mg/d intravenously, followed with 50 g/d) and ganciclovir (0.075 g administered intravenously daily). On November 15, the patient’s symptoms had improved, and she was successfully extubated. High-flow oxygen therapy was continued, and the chest X-ray indicated that lung lesions were largely resolved (Figure 3E). The patient was transferred to a local hospital for continued treatment 1 week later.

## 3 Discussion

*Legionella* is an environmental microbe that exists in various water bodies (e.g., lakes, streams, and artificial reservoirs), and was first identified after an outbreak of infection at an American veterans’ rally in 1976 (Wang et al., 2019). The pathogen is a Gram-negative facultative intracellular bacterium primarily transmitted through the inhalation of infectious aerosols, with an incubation period ranging from 2 to 14 days (Leenheer et al., 2023). *Legionella* genus comprises 58 species and 3 subspecies, with *L pneumophila* serotype 1 accounting for more than 80% of all *Legionella* spp. Infections. Serotypes 1, 4, and 6 are the most commonly isolated from patients with severe community-acquired pneumonia (Yu et al., 2002). Other *Legionella* spp., such as *Legionella* micdadei, *Legionella* bozemanae, *Legionella* longbeachae, and *Legionella* dumoffii, account for the remaining 10% of human cases of *Legionella* pneumonia (Chambers et al., 2021). Compared to other atypical respiratory pathogens, *Legionella* pneumonia progresses more rapidly and clinical diagnosis remains challenging. Early diagnosis using NGS technology and precise antibiotic treatment can significantly enhance patient clinical outcomes (Li et al., 2022). Studies have demonstrated that, in comparison to metagenomic next-generation sequencing (mNGS) technology, which is cost-prohibitive and unable to concurrently detect both DNA and RNA, targeted tNGS offers superior speed, cost-effectiveness, and accuracy (Hennebique et al., 2017). Consequently, tNGS holds significant potential for the detection of atypical respiratory pathogens. Our patients presented with severe pneumonia and exhibited a poor response to empirical anti-infective treatment. *Legionella pneumophila* was detected using tNGS of BALF, and the patient’s condition improved after targeted treatment. As an intracellular pathogen, *Legionella* poses challenges, as conventional *in vitro* drug susceptibility tests may not accurately predict *in vivo* efficacy, Therefore, antibacterial agents must demonstrate adequate bactericidal activity both *in vitro* and *in vivo*, along with effective intracellular penetration, to successfully treat Legionnaires’ disease. Currently, fluoroquinolones and macrolides are considered the first-line therapies for *Legionella* pneumonia (Viasus et al., 2022). However, the clinical application of these drugs is limited by issues such as drug resistance and adverse reactions, including gastrointestinal symptoms, hepatotoxicity, and arrhythmia (Bruin et al., 2014; Stahlmann and Lode, 2010). Patient 2 experienced intolerable limb twitching and hand shaking during moxifloxacin treatment leading to switching drug therapy to a second-line agent. These limitations of the first-line treatments have prompted the search for new treatment strategies.

Omadacycline is a semi-synthetic antibacterial agent derived from minocycline, capable of being administered both intravenously and orally. It demonstrates strong antibacterial activity against Gram-positive, Gram-negative, atypical and anaerobic bacteria (Zhanel et al., 2020). Omadacycline has a large distribution volume of 190 L and a low protein binding rate of21.3%, allowing for extensive distribution throughout the body. Among the new generation of tetracyclines, Omadacycline achieves higher concentration in alveolar macrophages (AM) and alveolar epithelial inner fluid (ELF) compared to tigecycline, making it particularly suitable for the treatment of lung infection (Gotfried et al., 2017). However, data on the efficacy of Omadacycline in severe *Legionella* pneumonia remain limited. In a clinical trial, omadacycline was found to be comparable to moxifloxacin, achieving an 87% early clinical success rate among 37 individuals with *L. pneumophila* pneumonia (Stets et al., 2019). Lu et al. reported a case of a patient with *L. pneumophila* pneumonia caused by drowning, who was successfully treated with omadacycline (Lu et al., 2023). Lv et al. reported a case of severe *L. pneumophila* pneumonia complicated with multiple organ dysfunction, which was also successfully treated with omadacycline (Lv et al., 2024). Zhu et al. reported successful treatment with omadacycline after experiencing abnormal liver function in patients with *L. pneumophila* pneumonia (Zhu et al., 2024). The current study reported three patients with severe *L. pneumophila* pneumonia with improved clinical outcomes after switching to omadacycline following a lack of response to empirical treatment with moxifloxacin. Omadacycline demonstrated relative intracellular penetrance against *L. pneumophila* serotype 1, effectively killing the bacteria, and showed either stronger or comparable *in vitro* activity relative to similar antibiotics (MIC90: omadacycline 0.25 vs doxycycline 1 vs azithromycin 0.5 vs moxifloxacin 0.016) (Dubois et al., 2020). In addition, the drug is not metabolized and is excreted by the feces (81.1%) and the kidney (14.4%) in prototype form, suggesting that no dose adjustment is necessary for patients with hepatic and renal insufficiency, thereby making it particularly suitable for special populations (Karlowsky et al., 2019; Berg et al., 2018; Kovacs et al., 2020). Both patient 2 and patient 3 were elderly patients with renal insufficiency requiring intermittent dialysis and who received omadacycline without dose adjustment. No adverse reactions were observed in this study following omadacycline administration, demonstrating its safety advantages.

In this study, the initial response of the 3 patients to moxifloxacin was suboptimal, raising concerns about the potential for drug resistance. Typically, pathogens can develop resistance during treatment, and exposure to antibiotics may also accelerate this process. The origin of antimicrobial resistance in clinical isolates remains to be elucidated. Possible explanations include the acquisition of a resistant *L. pneumophila* strain from the hospital environment, or the emergence of resistant mutations during moxifloxacin treatment (Bruin et al., 2014). Reports of quinolone-resistant clinical isolates are limited. Studies indicate that drug resistance mutations may occur in individuals with *Legionella* infection, leading to treatment failure during quinolone therapy. The mechanism of drug resistance may be related to mutations in the gyrA (83 amino acid) region of *Legionella* (Shadoud et al., 2015). Both patient 2 and patient three were diagnosed with ANCA-associated vasculitis resulting in renal injury and were undergoing intermittent dialysis while receiving long-term immunosuppressive therapy. We speculate that the occurrence of drug resistance of *L. pneumophila* may be related to the use of moxifloxacin in patients with recurrent infections or to insufficient concentrations due to low protein levels in patients with renal insufficiency. In recent years, strains of *Legionella* isolated from the environment have been found to exhibit resistance to therapeutic drugs both domestically and internationally. *Legionella* is often associated with severe and critical illness, especially in immuno-compromised people. Domestic studies showed that a total of 149 strains of *L. pneumophila* serotype 1, of which 25 strains were resistant to azithromycin, resulting in a resistance rate of 16.78% (25/149) (Jia et al., 2019). All strains were sourced from the environment, and the expression of the lpeAB gene, which encodes an efflux pump, is responsible for the decreased sensitivity of these 25 strains to azithromycin. Researchers in Poland identified a non-serotype 1 strain of *L. pneumophila* isolated from the water system in a sanitorium that displayed resistance to azithromycin and reduced sensitivity to ciprofloxacin and rifampicin (Sikora et al., 2017). The geographic variability in drug resistance among *Legionella* strains underscores the practical significance of understanding the drug susceptibility data across different regions to guide local clinical drug use (Zhao et al., 2021). Antimicrobial resistance of clinical isolates of *Legionella* spp. Has yet to be documented in China. The climate in South China, characterized by warm temperatures, high precipitation and elevated humidity, is conducive to the growth of *Legionella*. Future research will focus on assessing the status of *Legionella* infections and drug resistance in this region, especially among immunosuppressed populations.

## 4 Conclusion

This study reports three patients with severe *L. pneumophila* pneumonia who were successfully treated with omadacycline as a second-line therapy after initial treatment with moxifloxacin proved ineffective or was poorly tolerated. Omadacycline is a novel 9-aminomethyclic antibiotic that may serve as a first-line treatment option for severe *L. pneumophila* infections or as a second-line option after moxifloxacin treatment failure, especially in patients with liver and kidney dysfunction or quinolone intolerance. However, further clinical evidence is necessary to substantiate its efficacy. In future research, we will focus on exploring the prevalence of *Legionella* infections and drug resistance in this region, with a particular emphasis on the immunosuppressed population.

## Data Availability

The raw data supporting the conclusions of this article will be made available by the authors, without undue reservation.
